# Supplementary material for: Evaluation of diagnostic measurements in patients with non-specific complaints: A secondary analysis after implementation of a care-pathway in the emergency department
Source: PLoS One. 2025 Sep 9;20(9):e0331060. doi: 10.1371/journal.pone.0331060 (PMC12419613; doi:10.1371/journal.pone.0331060)
Supplement: Attachment — Submitted filename: Review comment_250522.pdf [file pone.0331060.s001.pdf]

Review comment: "**Evaluation of diagnostic measurements in patients with non-specific complaints: a secondary analysis after implementation of a care-pathway in the Emergency Department"**

This study addresses an important and timely issue in emergency medicine, optimizing the diagnostic approach for patients presenting with non-specific complaints. Given the growing number of older and medically complex patients in the ED, the evaluation of structured diagnostic pathways is highly relevant. The authors tackle a clinically significant challenge and contribute to an area where evidence is still evolving, making this research both valuable and of broad interest to clinicians and researchers alike.

The aim of the study, to assess feasibility, identify unnecessary tests, and evaluate the added value of the NSC care pathway, is clearly stated and relevant. However, the statistical approach, while appropriate for descriptive comparisons (i.e., counts, percentages, chi-square/Fisher's exact tests), appears limited in its ability to fully address these broader evaluative goals. To strengthen the analysis, the authors might consider including:

- Multivariable analysis to adjust for potential confounders (age, sex, comorbidity, triage level) when comparing testing patterns or outcomes.
- Logistic regression to assess whether specific diagnostic tests were independently associated with relevant clinical outcomes (diagnosis accuracy, admission, return visits).
- If resource utilization is a focus, cost-effectiveness or time-to-diagnosis analyses could also enhance the study's impact. Did it speed up decisions? Shorten length of stay in the ED? Reduced unnecessary admissions?

Adding these elements could provide a more robust evaluation of the pathway's effectiveness and clinical relevance beyond adherence and test frequency.

Introduction

1. The authors present the background and current knowledge gaps clearly and effectively. The introduction outlines the clinical relevance of the topic and highlights important limitations in existing research, particularly regarding diagnostic challenges in patients with non-specific symptoms. This framing makes the rationale for the study compelling and underscores the potential contribution of the current work to the field of geriatric emergency care.

2. The manuscript refers to a previous analysis, which may limit its clarity as a stand-alone article. I recommend rephrasing or expanding this section to ensure the study is fully self-contained and understandable without requiring access to prior publications.

Methods

3. I recommend expanding the "Design and Setting" section to provide additional context about the two hospitals. For example, indicating whether the hospitals are located in urban or rural areas, whether their EDs operate 24/7, and including approximate bed counts or annual ED visit volumes would help readers better understand the study setting and its generalizability. Is the ED

4. Please clarify the staffing structure of the emergency departments included in the study. Are the EDs primarily staffed by emergency physicians, or do physicians from other specialties (e.g., internal medicine, surgery) manage patients? This information would help readers better understand the clinical context and how it may influence diagnostic decision-making and patient management.

5. All tables are easy to follow and structured in a good way.

6. Could you clarify the rationale for including chest X-rays as a routine component of the NSC care pathway? Given current evidence suggesting that CXRs has no diagnostic value in patients without respiratory signs or symptoms, if a reliable medical history can be obtained. Unnecessary routine CXRs delays the ED flow and increases healthcare costs. Additionally, the growing use of point-of-care ultrasound (POCUS) as a rapid, low-risk alternative warrants discussion, particularly in geriatric emergency care where minimizing delays is critical. Imaging can often wait until after admission.

7. The current analysis offers a solid descriptive overview of diagnostic testing in patients with non-specific complaints. However, incorporating additional analyses such as multivariable adjustment, diagnostic yield comparisons, or associations between specific tests and clinical outcomes would allow the study to move beyond a descriptive audit toward a more rigorous evaluation of the care pathway's effectiveness, efficiency, and clinical value. Such refinements would strengthen the manuscript and provide greater insight into the practical impact of the implemented diagnostic strategy.

<u>Results</u>

8. The description of inclusion periods for control and intervention groups across the two hospitals is difficult to follow due to formatting and repetitive structure. Consider rephrasing this section using consistent and clear date formats and separating the information for each hospital into distinct sentences to improve readability.

   "Hospital 1 included 128 control patients between 1-8-2020 and 31-3-2021 and 212 intervention patients between 1-4-2021 and 30-11-2024. Hospital 2 included 36 control patients between 1-12-2020 and 31-8-2021 and 23 intervention patients from 1-9-2021 until 30-11-2024."

   "Hospital 1 included 128 control patients between August 1, 2020, and March 31, 2021, and 212 intervention patients between April 1, 2021, and November 30, 2024. Hospital 2 included 36 control patients between December 1, 2020, and August 31, 2021, and 23 intervention patients between September 1, 2021, and November 30, 2024."

9. It would strengthen the manuscript to include key baseline characteristics such as age, gender, and comorbidity burden for the study population. This information would help readers better understand the patient group studied and assess the generalizability of the findings.

Discussion

10. The discussion section is well-written, clearly structured, and easy to follow. It effectively contextualizes the findings within existing literature and highlights the study’s clinical relevance.

11. The recommendation to continue standardized use of chest X-rays would benefit from additional context. It would be valuable to briefly address the ongoing discussion in the literature regarding the limited impact of chest X-rays on clinical management in patients, and the growing interest in alternatives such as point-of-care ultrasound. This would help justify the inclusion of chest X-ray in the care pathway and align the discussion with current clinical debates.

    “We also recommend to incorporate bladder scans into routine care, and continuing the standardized use of chest X-rays and urinalysis.”
