# Supplementary material for: Evaluation of diagnostic measurements in patients with non-specific complaints: A secondary analysis after implementation of a care-pathway in the emergency department
Source: PLoS One. 2025 Sep 9;20(9):e0331060. doi: 10.1371/journal.pone.0331060 (PMC12419613; doi:10.1371/journal.pone.0331060)
Supplement: Attachment — Submitted filename: Response to reviewers.docx [file pone.0331060.s003.docx]

**Point to point response**

**Editor:**

**Dear Dr. van der Velde,
Thank you for submitting your manuscript to PLOS ONE. After careful consideration, we feel that it has merit but does not fully meet PLOS ONE’s publication criteria as it currently stands. Therefore, we invite you to submit a revised version of the manuscript that addresses the points raised during the review process.**

*Dear Dr. Bayer,*

*We thank you for your effort to assess our manuscript. We greatly appreciate the constructive insights that have contributed to its improvement.*

*In response to your and the reviewers’ comments, we have revised the manuscript accordingly, with all changes highlighted using track changes. We have also provided a detailed, point-by-point reply addressing each of your concerns.*

*In particular, we have expanded the methods section to enhance this article as a stand-alone piece. We added details about the participating hospitals, emergency departments, and the criteria for nonspecific complaints (NSC) to ensure the study is fully self-contained and its results can be interpreted in light of these characteristics. Additionally, baseline characteristics were included to provide better insight into the study population.*

*We took note of the insightful comments regarding our recommended diagnostic work-up. Specifically, concerning the role of ECG, we acknowledge that a 16% rate of clinically significant abnormalities is notable and supports the routine use of ECG in this population. Regarding urinalysis, we appreciate the complexity of diagnosing urinary tract infections (UTIs) in older adults, especially those with NSC. While urinalysis contributed to detecting true infections, we emphasize that a positive urinalysis alone should not lead to antibiotic treatment without corroborating clinical signs or symptoms. We have clarified that urinalysis should be integrated with comprehensive clinical assessment and culture results to avoid overtreatment and unnecessary antibiotic use. This nuanced approach better reflects current evidence and clinical practice challenges in this population.*

*We believe that the revised manuscript is now suitable for publication in European Geriatric Medicine.*

*On behalf of all authors,*

*Marleen van der Velde*

Comment 1.1: In particular, would you address the important observations made by Reviewer 2, especially your conclusions about urine analysis and ECG.

*Reply 1.1: We thank Reviewer 2 for their insightful observations regarding our conclusions on urine analysis and ECG use in older adults presenting with nonspecific complaints (NSC). We have carefully reconsidered these aspects in light of their comments and revised our manuscript accordingly.*

*Regarding ECG, we acknowledge that our original wording may have understated its diagnostic value. With 16% of patients showing clinically significant abnormalities, this is indeed a notable yield—approximately one in six patients. Given the non-invasive nature, low cost, and potential to uncover actionable cardiac pathology, we now support the routine use of ECG in the initial assessment of NSC patients. This approach can facilitate earlier recognition of serious conditions that might otherwise remain undetected, especially considering the often vague presentations and challenges in history taking in this population.*

*Concerning urine analysis, we appreciate the complexity of diagnosing urinary tract infections (UTIs) in older adults, especially those with NSC, due to the high prevalence of asymptomatic bacteriuria and atypical presentations. While our data show that urinalysis contributed to detecting true infections, we emphasize that urinalysis alone should not dictate antibiotic treatment. Clinical judgment, additional signs or symptoms, and culture results must inform the decision-making process to avoid unnecessary antibiotic use and potential harm. We have clarified this balance in the discussion to stress that while urinalysis can be a valuable component of the diagnostic work-up, it should be interpreted within the broader clinical context rather than used as a standalone test.*

*We believe these clarifications better reflect the diagnostic challenges and nuances of managing NSC in older adults and strengthen our recommendations. The relevant manuscript sections have been updated to reflect these points (see pages 18-20, lines 329-342 and 370-378): ‘**Urinalysis, performed more frequently in the intervention group, detected true infections in about 20% of cases. In nearly 10%, antibiotics were prescribed for what appeared to be asymptomatic bacteriuria. While clinical judgment likely informed these decisions, we did not monitor whether other signs of infection were present. Diagnosing a urinary tract infection (UTI) in older adults is complex, particularly in patients with NSC, as presentations are often atypical, such as confusion, falls, or reduced appetite, rather than classic urinary symptoms (24). Genitourinary infections are among the most frequent diagnoses in this group, which may justify performing urinalysis as part of the diagnostic work-up (3, 25). However, given the high prevalence of asymptomatic bacteriuria and the risk of false positives, urinalysis should always be interpreted in the broader clinical context (26). While our findings support routine urinalysis in this group, treatment decisions should not rely solely on these results. Instead, urinalysis should be one component of the overall diagnostic reasoning, helping to assess the likelihood of a UTI. It is also important to wait for culture results and consider alternative explanations for symptoms to avoid unnecessary antibiotic use. […] Our findings showed that 16% of NSC-patients showed significant abnormalities on ECG. This indicates that the diagnostic yield of ECG in this population is relatively high, with the test revealing a potentially important abnormality in approximately 1 out of every 6 patients. Given the often vague and non-specific nature of NSC presentations and the frequent unreliability of history taking in this population, the ECG appears to be a valuable diagnostic tool that can uncover underlying cardiac conditions that might otherwise remain undetected. These results support the routine use of ECG in the initial assessment of patients with NSC, as it may contribute to earlier recognition of serious pathologies and guide further diagnostic or therapeutic steps.’*

Comment 1.2: You may feel that the suggestions for further analysis of additional outcomes made by Reviewer 1 would best be considered in a separate paper.

*Reply 1.2: We agree that the suggestions for further analysis of additional outcomes raised by Reviewer 1 are valuable. However, given the exploratory nature of the study and the retrospective data collection in the control group, we chose not to include multivariable or regression analyses, as these could imply a level of causal inference that the study is not designed to support.*

Comment 1.3: Can you also check the phrasing on lines 166-169 that I found a bit confusing.

*Reply 1.3: Thank you for pointing this out. We have revised the phrasing on lines 166–169 to enhance clarity and readability. See page 13, lines 216-221. ‘Although glucose and alanine aminotransferase (ALAT) already had high completion rates in the control group, they were performed significantly more often in the intervention group (ALAT 98.6% vs. 93.9%, p < 0.001; glucose 99.6% vs 93.9%, p=0.001)’*

Comment 1.4: The references also need reviewing to ensure they are complete - I spotted missing page numbers (ref 1), incorrect first author (ref 19) and lack of consistency of citing full or abbreviated journal names (e.g. refs 1 & 2). Has reference 12 now ben published?

*Reply 1.4: Thank you for your careful review of the references. We have thoroughly checked and updated all references to ensure completeness and consistency. Page numbers have been added where missing (e.g., ref 1), author details have been corrected (e.g., ref 19), and journal names have been made consistent throughout. Reference 12 has now been published, and the citation has been updated accordingly.*

**Reviewer #1:**

**This study addresses an important and timely issue in emergency medicine, optimizing the diagnostic approach for patients presenting with non-specific complaints. Given the growing number of older and medically complex patients in the ED, the evaluation of structured diagnostic pathways is highly relevant. The authors tackle a clinically significant challenge and contribute to an area where evidence is still evolving, making this research both valuable and of broad interest to clinicians and researchers alike.**

*Dear reviewer,*

*We thank you for your effort to assess our manuscript. We greatly appreciate the constructive insights that have contributed to its improvement.*

*In response to your and the reviewers’ comments, we have revised the manuscript accordingly, with all changes highlighted using track changes. We have also provided a detailed, point-by-point reply addressing each of your concerns.*

*In particular, we have expanded the methods section to enhance this article as a stand-alone piece. We added details about the participating hospitals, emergency departments, and the criteria for nonspecific complaints (NSC) to ensure the study is fully self-contained and its results can be interpreted in light of these characteristics. Additionally, baseline characteristics were included to provide better insight into the study population.*

*We took note of the insightful comments regarding our recommended diagnostic work-up. Specifically, concerning the role of ECG, we acknowledge that a 16% rate of clinically significant abnormalities is notable and supports the routine use of ECG in this population. Regarding urinalysis, we appreciate the complexity of diagnosing urinary tract infections (UTIs) in older adults, especially those with NSC. While urinalysis contributed to detecting true infections, we emphasize that a positive urinalysis alone should not lead to antibiotic treatment without corroborating clinical signs or symptoms. We have clarified that urinalysis should be integrated with comprehensive clinical assessment and culture results to avoid overtreatment and unnecessary antibiotic use. This nuanced approach better reflects current evidence and clinical practice challenges in this population.*

*We believe that the revised manuscript is now suitable for publication in European Geriatric Medicine.*

*On behalf of all authors,*

*Marleen van der Velde*

Comment 2.1: The aim of the study, to assess feasibility, identify unnecessary tests, and evaluate the added value of the NSC care pathway, is clearly stated and relevant. However, the statistical approach, while appropriate for descriptive comparisons (i.e., counts, percentages, chi-square/Fisher’s exact tests), appears limited in its ability to fully address these broader evaluative goals. To strengthen the analysis, the authors might consider including:
- Multivariable analysis to adjust for potential confounders (age, sex, comorbidity, triage level) when comparing testing patterns or outcomes.
- Logistic regression to assess whether specific diagnostic tests were independently associated with relevant clinical outcomes (diagnosis accuracy, admission, return visits).
- If resource utilization is a focus, cost-effectiveness or time-to-diagnosis analyses could also enhance the study’s impact. Did it speed up decisions? Shorten length of stay in the ED? Reduced unnecessary admissions?
Adding these elements could provide a more robust evaluation of the pathway’s effectiveness and clinical relevance beyond adherence and test frequency.

*Reply 2.1: We appreciate the thoughtful suggestions to strengthen the analytical approach. However, the primary aim of this study was to explore the feasibility and practical implementation of the NSC care pathway, with a focus on descriptive evaluation of test use, adherence, and basic clinical outcomes. Given the exploratory nature and the study design—particularly the retrospective data collection in the control group—we chose not to include multivariable or regression analyses, as these could imply a level of causal inference that the study is not designed to support. We agree that further research using prospective or interventional designs would be well-suited to address these important questions regarding clinical effectiveness, resource use, and cost-effectiveness.*

Comment 2.2: The authors present the background and current knowledge gaps clearly and effectively. The introduction outlines the clinical relevance of the topic and highlights important limitations in existing research, particularly regarding diagnostic challenges in patients with non-specific symptoms. This framing makes the rationale for the study compelling and underscores the potential contribution of the current work to the field of geriatric emergency care.

*Reply 2.2: Thank you for your positive feedback. We are pleased to hear that the introduction effectively presented the clinical relevance and research gaps, and that the rationale for our study was clear.*

Comment 2.3: The manuscript refers to a previous analysis, which may limit its clarity as a stand-alone article. I recommend rephrasing or expanding this section to ensure the study is fully self-contained and understandable without requiring access to prior publications.

*Reply 2.3: Thank you for this suggestion. We have revised the method section to reduce reliance on the previous study and to ensure that the manuscript stands on its own. Additional context and clarifying details have been added, see page 5-6, lines 99-123. ‘The study included patients aged 70 and older who presented to the ED with NSC and met specific inclusion criteria. These criteria included referrals by general practitioners or elderly care physicians during weekday working hours, or triage in the ED with NSCs requiring ED admission. NSC were divided into five referral categories: 1) somatic problems, such as weakness, not feeling well, change in nutritional status or unexplained weight loss; 2) an increased demand of care, such as loss of independency, a necessity for starting home-care or for change in the living situation (such as admission to a care-home), not indicated previously; 3) cognitive problems, such as disorientation, changes in behavior, abrupt cognitive decline; 4) a decline in functional status, such as loss of mobility; and 5) unexplained falls, not related to extrinsic factors.*

*NSC care-pathway*

*The detailed trial protocol, including specifics of the care-pathway, has been published previously (7, 12). Before the implementation of the care pathway, patients received standard care. Upon arrival, they underwent triage, followed by history taking and physical examination. Frailty screening was not routinely performed, and there was no standardized approach for screening across the multiple domains of the comprehensive geriatric assessment (CGA); both were left to the discretion of the attending physician. Diagnostic tests were ordered according to the physician’s and nursing staff's judgment.*

*In contrast, the care pathway offered standardized care for patients with NSC (7). Upon their arrival at the ED, patients were triaged, and assessed for frailty in both cognitive as functional domains using the APOP-screener (13). In addition to solely somatic history taking, physicians conducted screening across various domains in line with the CGA (14). NSC-patients received standard diagnostic measurements, including an electrocardiogram, urinalysis, chest radiography, bladder ultrasound and comprehensive blood tests (table 1). Further diagnostic tests could be performed according to the physician’s choice.’*

Comment 2.4: I recommend expanding the "Design and Setting" section to provide additional context about the two hospitals. For example, indicating whether the hospitals are located in urban or rural areas, whether their EDs operate 24/7, and including approximate bed counts or annual ED visit volumes would help readers better understand the study setting and its generalizability.

*Reply 2.4: Thank you for this helpful suggestion. We have expanded the "Design and Setting" section to include additional context about both hospitals. See page 5, lines 87-98. ‘Hospital 1 provides 24/7 ED service with only occasional presentation stops and handles approximately 20,500 ED visits annually. The ED houses 19 operational bed during office-hours and 15 operational beds during evening and nights. Emergency Physicians (EP) and internal medicine residents are 24/7 present and both are involved in the care of ED patients. Internists are present during office hours and available on call during evenings and nights. Hospital 2, with a lower annual ED volume of about 13,500 visits, also offers 24/7 ED service with infrequent presentation stops. The ED includes seven operational beds and four additional observation beds, intended for patient monitoring rather than admission, and supervised by the EP. EPs are present during the day and evening shifts, while at night they are on call.’*

Comment 2.5: Please clarify the staffing structure of the emergency departments included in the study. Are the EDs primarily staffed by emergency physicians, or do physicians from other specialties (e.g., internal medicine, surgery) manage patients? This information would help readers better understand the clinical context and how it may influence diagnostic decision-making and patient management.

*Reply 2.5: Thank you for this helpful suggestion. We have expanded the "Design and Setting" section to include additional context about the staffing of the ED’s. See page 5, lines 87-98. ‘Hospital 1 provides 24/7 ED service with only occasional presentation stops and handles approximately 20,500 ED visits annually. The ED houses 19 operational bed during office-hours and 15 operational beds during evening and nights. Emergency Physicians (EP) and internal medicine residents are 24/7 present and both are involved in the care of ED patients. Internists are present during office hours and available on call during evenings and nights. Hospital 2, with a lower annual ED volume of about 13,500 visits, also offers 24/7 ED service with infrequent presentation stops. The ED includes seven operational beds and four additional observation beds, intended for patient monitoring rather than admission, and supervised by the EP. EPs are present during the day and evening shifts, while at night they are on call.’*

Comment 2.5: All tables are easy to follow and structured in a good way.

*Reply 2.5: Thank you for your positive feedback.*

Comment 2.6: Could you clarify the rationale for including chest X-rays as a routine component of the NSC care pathway? Given current evidence suggesting that CXRs has no diagnostic value in patients without respiratory signs or symptoms, if a reliable medical history can be obtained. Unnecessary routine CXRs delays the ED flow and increases healthcare costs. Additionally, the growing use of point-of-care ultrasound (POCUS) as a rapid, low-risk alternative warrants discussion, particularly in geriatric emergency care where minimizing delays is critical. Imaging can often wait until after admission.

*Reply 2.6: Thank you for raising this important point. We agree that the routine use of chest X-rays (CXR) should be carefully considered, particularly in the context of efficient ED care. However, in patients presenting with non-specific complaints (NSC), obtaining a reliable medical history is often difficult or impossible—especially in older adults with cognitive impairment or communication barriers. In such cases, a CXR can support the identification of otherwise missed diagnoses, such as pneumonia or heart failure, which may present atypically.*

*We acknowledge that several studies have questioned the diagnostic value of routine chest X-rays, particularly when no respiratory symptoms are reported. However, these studies often excluded patients with unreliable or incomplete medical histories, thereby limiting their relevance to our study population. Given the diagnostic uncertainty that frequently characterizes NSC presentations, especially in vulnerable older adults, we believe that a standardized approach including CXR remains justified.*

*We also recognize the potential of point-of-care ultrasound (POCUS) as a rapid and low-risk alternative. However, at the time of the care pathway’s implementation, POCUS was not yet fully integrated into routine practice at the participating EDs. We acknowledge its growing role in geriatric emergency care and have added a note on its potential and future application in the discussion. We have added these considerations into the revised Discussion (see page 19, lines 343-356): ‘The diagnostic value of chest X-rays in the ED remains a topic of debate. While several studies have questioned the usefulness of routine chest X-rays—particularly in the absence of respiratory symptoms—these studies often excluded patients with unreliable or incomplete medical histories, such as those with cognitive impairment (24, 25). As a result, their findings are less applicable to older adults presenting with NSC, in whom history taking is frequently limited. In such cases, chest X-rays can help detect otherwise unrecognized conditions like pneumonia or heart failure, which often present atypically. Given the diagnostic uncertainty and broad differential diagnoses characteristic of NSC presentations, a standardized approach that includes chest X-rays may contribute to more accurate and timely diagnoses. Point-of-care ultrasound (POCUS) is increasingly recognized as a rapid, low-risk diagnostic tool with significant potential in geriatric emergency care (26, 27). However, at the time the care pathway was implemented, POCUS had not yet been fully integrated into routine practice at the participating EDs. Its growing adoption warrants consideration in future care protocols, as it may offer a valuable alternative to chest X-rays in the evaluation of older adults with NSC.’*

Comment 2.7: The current analysis offers a solid descriptive overview of diagnostic testing in patients with non-specific complaints. However, incorporating additional analyses such as multivariable adjustment, diagnostic yield comparisons, or associations between specific tests and clinical outcomes would allow the study to move beyond a descriptive audit toward a more rigorous evaluation of the care pathway’s effectiveness, efficiency, and clinical value. Such refinements would strengthen the manuscript and provide greater insight into the practical impact of the implemented diagnostic strategy.

*Reply 2.7: We appreciate your thoughtful suggestions. As this study was primarily designed as a feasibility and implementation evaluation, we focused on descriptive analyses to explore adherence and test utilization patterns within the care pathway. While we agree that additional analyses—such as multivariable adjustments or outcome associations—could offer deeper insights, the current dataset and study design were not optimized for such analyses without risking overinterpretation.*

Comment 2.8: The description of inclusion periods for control and intervention groups across the two hospitals is difficult to follow due to formatting and repetitive structure. Consider rephrasing this section using consistent and clear date formats and separating the information for each hospital into distinct sentences to improve readability. “Hospital 1 included 128 control patients between 1-8-2020 and 31-3-2021 and 212 intervention patients between 1-4-2021 and 30-11-2024. Hospital 2 included 36 control patients between 1-12-2020 and 31-8-2021 and 23 intervention patients from 1-9-2021 until 30-11-2024.”
“Hospital 1 included 128 control patients between August 1, 2020, and March 31, 2021, and 212 intervention patients between April 1, 2021, and November 30, 2024. Hospital 2 included 36 control patients between December 1, 2020, and August 31, 2021, and 23 intervention patients between September 1, 2021, and November 30, 2024.”

*Reply 2.8: We have revised the text to use consistent date formats and have restructured the section by describing each hospital’s inclusion periods in separate sentences to improve clarity and readability.. See page 10, lines 188-193. ‘Hospital 1 included 128 control patients between 1-8-August 1, 2020 and 31-3-March 31, 2021 and 212 intervention patients between April 1,1-4-2021 and 30-11-November 30, 2024. Hospital 2 included 36 control patients between 1-12-December 1, 2020 and 31-8-August 31, 2021 and 23 intervention patients from 1-9-September 1, 2021 until 30-11-November 30, 2024. In total, 399 patients were included in the participating hospitals, comprising 164 control patients and 235 intervention patients.’*

Comment 2.9: It would strengthen the manuscript to include key baseline characteristics such as age, gender, and comorbidity burden for the study population. This information would help readers better understand the patient group studied and assess the generalizability of the findings.

*Reply 2.9: Thank you for this suggestion. We have now included key baseline characteristics—such as age, gender, and comorbidity burden—in the manuscript to provide a clearer understanding of the study population. See page 10-11, lines 195-201. ‘Baseline characteristics are presented in table 2. Age and sex was comparable between both control and intervention groups. Significant differences were found between the control and intervention group. Patients in the intervention group were more likely to use multiple medications, with 71.1% using five or more compared to 50.6% in the control group (p<0.001). They were also more frequently assigned lower triage levels (U3–U5: 70.4% vs. 56.8%, p<0.001). The most common reason for referral in the intervention group was somatic problems (47.7%), followed by cognitive issues (22.1%).’’*

Comment 2.10: The discussion section is well-written, clearly structured, and easy to follow. It effectively contextualizes the findings within existing literature and highlights the study’s clinical relevance.

*Reply 2.10: Thank you for your positive feedback.*

Comment 2.11: The recommendation to continue standardized use of chest X-rays would benefit from additional context. It would be valuable to briefly address the ongoing discussion in the literature regarding the limited impact of chest X-rays on clinical management in patients, and the growing interest in alternatives such as point-of-care ultrasound. This would help justify the inclusion of chest X-ray in the care pathway and align the discussion with current clinical debates. “We also recommend to incorporate bladder scans into routine care, and continuing the standardized use of chest X-rays and urinalysis.”

*Reply 2.11: Thank you for raising this important point. We agree that the routine use of chest X-rays (CXR) should be carefully considered, particularly in the context of efficient ED care. However, in patients presenting with non-specific complaints (NSC), obtaining a reliable medical history is often difficult or impossible—especially in older adults with cognitive impairment or communication barriers. In such cases, a CXR can support the identification of otherwise missed diagnoses, such as pneumonia or heart failure, which may present atypically.*

*We acknowledge that several studies have questioned the diagnostic value of routine chest X-rays, particularly when no respiratory symptoms are reported. However, these studies often excluded patients with unreliable or incomplete medical histories, thereby limiting their relevance to our study population. Given the diagnostic uncertainty that frequently characterizes NSC presentations, especially in vulnerable older adults, we believe that a standardized approach including CXR remains justified.*

*We also recognize the potential of point-of-care ultrasound (POCUS) as a rapid and low-risk alternative. However, at the time of the care pathway’s implementation, POCUS was not yet fully integrated into routine practice at the participating EDs. We acknowledge its growing role in geriatric emergency care and have added a note on its potential and future application in the discussion. We have added these considerations into the revised Discussion (see page 18-19, lines 343-356): ‘The diagnostic value of chest X-rays in the ED remains a topic of debate. While several studies have questioned the usefulness of routine chest X-rays—particularly in the absence of respiratory symptoms—these studies often excluded patients with unreliable or incomplete medical histories, such as those with cognitive impairment (24, 25). As a result, their findings are less applicable to older adults presenting with NSC, in whom history taking is frequently limited. In such cases, chest X-rays can help detect otherwise unrecognized conditions like pneumonia or heart failure, which often present atypically. Given the diagnostic uncertainty and broad differential diagnoses characteristic of NSC presentations, a standardized approach that includes chest X-rays may contribute to more accurate and timely diagnoses. Point-of-care ultrasound (POCUS) is increasingly recognized as a rapid, low-risk diagnostic tool with significant potential in geriatric emergency care (26, 27). However, at the time the care pathway was implemented, POCUS had not yet been fully integrated into routine practice at the participating EDs. Its growing adoption warrants consideration in future care protocols, as it may offer a valuable alternative to chest X-rays in the evaluation of older adults with NSC.’*

**Reviewer #2: This is an interesting study on a very important topic and directly relevant to clinical practice. I have the following minor recommendations:**

*Dear reviewer,*

*We thank you for your effort to assess our manuscript. We greatly appreciate the constructive insights that have contributed to its improvement.*

*In response to your and the reviewers’ comments, we have revised the manuscript accordingly, with all changes highlighted using track changes. We have also provided a detailed, point-by-point reply addressing each of your concerns.*

*In particular, we have expanded the methods section to enhance this article as a stand-alone piece. We added details about the participating hospitals, emergency departments, and the criteria for nonspecific complaints (NSC) to ensure the study is fully self-contained and its results can be interpreted in light of these characteristics. Additionally, baseline characteristics were included to provide better insight into the study population.*

*We took note of the insightful comments regarding our recommended diagnostic work-up. Specifically, concerning the role of ECG, we acknowledge that a 16% rate of clinically significant abnormalities is notable and supports the routine use of ECG in this population. Regarding urinalysis, we appreciate the complexity of diagnosing urinary tract infections (UTIs) in older adults, especially those with NSC. While urinalysis contributed to detecting true infections, we emphasize that a positive urinalysis alone should not lead to antibiotic treatment without corroborating clinical signs or symptoms. We have clarified that urinalysis should be integrated with comprehensive clinical assessment and culture results to avoid overtreatment and unnecessary antibiotic use. This nuanced approach better reflects current evidence and clinical practice challenges in this population.*

*We believe that the revised manuscript is now suitable for publication in European Geriatric Medicine.*

*On behalf of all authors,*

*Marleen van der Velde*

Comment 3.1: I think it would be helpful to list the eligibility criteria for the NSC - I had to look this up in the research protocol which was time consuming yet the reader does need to know definitions of NSC to be able to interpret this paper.

Reply 3.1: *Thank you for this helpful suggestion. We agree that providing the eligibility criteria for NSC is important for clarity and independent interpretation. We have revised the Methods section accordingly to reduce reliance on the research protocol. Additional context and clarifying details have been added, see page 5-6, lines 100-108. ‘The study included patients aged 70 and older who presented to the ED with NSC and met specific inclusion criteria. These criteria included referrals by general practitioners or elderly care physicians during weekday working hours, or triage in the ED with NSCs requiring ED admission. NSC were divided into five referral categories: 1) somatic problems, such as weakness, not feeling well, change in nutritional status or unexplained weight loss; 2) an increased demand of care, such as loss of independency, a necessity for starting home-care or for change in the living situation (such as admission to a care-home), not indicated previously; 3) cognitive problems, such as disorientation, changes in behavior, abrupt cognitive decline; 4) a decline in functional status, such as loss of mobility; and 5) unexplained falls, not related to extrinsic factors.*

Comment 3.2: Please can you clarify what you mean by ‘normal or abnormal’ with respect to CT head - CT head is frequently abnormal in older patients but this is rarely acutely relevant in an ED situation - do you mean that there are new, acute changes or other chronic abnormal changes? The presence of acute changes directly relevant to the ED presentation is a significant finding so should be clarified as such - I would consider 21.3% of CT brains to have acute abnormalities to be very high in this setting, which could potentially alter your conclusions.

*Reply 3.2: Thank you for this valuable comment. We agree that clarification is needed. Head CT scans were classified as abnormal if they revealed new findings compared to previous imaging, acute pathology, or abnormalities with potential clinical relevance. Not all abnormalities were acute; some scans identified previously undetected chronic changes, such as old ischemic lesions, which may help explain the clinical presentation. We have added this into the method section, see page 9, lines 160-168. ‘Head CT scans were classified as normal or abnormal, with the specific indication for each scan documented. Scans were considered abnormal if they showed new findings compared to prior imaging, revealed acute pathology, or included abnormalities with potential clinical relevance. These findings were not always acute in nature and could also include previously undetected chronic conditions, such as old ischemic lesions identified for the first time.’*

*While 21.3% abnormal CT scans is relatively high, this is likely influenced by selection bias, as head CT was not part of the standardized protocol. Clinical indications—such as suspected intracranial bleeding or trauma—probably contributed to the more frequent use of CT in this group. Given this selection bias, we cannot recommend routine head CT as part of the care pathway. Instead, we suggest following established mild traumatic brain injury guidelines and reserving CT imaging for patients with unexplained new-onset cognitive or behavioral changes. This point has already been addressed in the discussion section, see page 20, lines 379-385. ‘CT imaging of the head was performed more frequently in the intervention group, suggesting that head CT was used when the diagnostic pathway did not clarify the clinical presentation. However, this increase should be interpreted with caution due to potential selection bias, as CT was not a standard protocol. Preexisting indications, such as suspected bleeding, may have influenced its more frequent use. Due to this selection bias, we cannot recommend routine inclusion of head CT in the care -pathway. We advise following mild traumatic brain injury guidelines and considering CT for unexplained new-onset cognitive or behavioral changes.’*

Comment 3.3: I am concerned that the paper recommends routine urinalysis in this population. Presence of a urinary pathogen does not necessarily constitute urinary tract infection (defined as ‘true infections’ in this paper) and may represent asymptomatic bacteria, of which there is a high prevalence in this population. I think your discussion suggests 30% of cases who had urinalysis received antibiotics? UTI should only be diagnosed in the presence of other clinical factors, not simply on urinalysis alone; even in non specific presentations there needs to be other signs or symptoms of UTI for diagnosis - are you able to elaborate if those that received antibiotics had them because of other signs or purely on urinalysis results? Please can the discussion reflect this: although there is some discussion around the risk of asymptomatic bacturia currently the paper recommends routine urinalysis - I do not feel recommending routine urinalysis for all patients is the natural conclusion of this paper without further study, and could potentially be harmful.

*Reply 3.3: Thank you for this valid concern. We do not know whether the 30% of patients who received antibiotics were treated solely based on urinalysis results, or whether there were additional clinical signs suggesting infection. It is reasonable to assume that clinical expertise informed these decisions and that treatment was not based on urinalysis alone, but we did not monitor this specifically. The distinction between asymptomatic bacteriuria and a true urinary tract infection (UTI) is indeed challenging in older adults. An abnormal urinalysis result should be accompanied by other clinical signs or symptoms to support a UTI diagnosis. However, this patient population presented to the ED for a reason—whether due to somatic issues, cognitive problems, or, for example, falls. In older adults, UTI often manifests atypically, with symptoms such as delirium, confusion, dizziness, drowsiness, falls, urinary incontinence, or poor appetite, often in the absence of fever or urinary complaints. This makes the diagnosis particularly difficult, especially in patients with non-specific complaints (NSC). Moreover, existing literature on NSC patients consistently shows genitourinary infections as the most common final diagnosis, highlighting how difficult it can be to either confirm or rule out this condition. We fully acknowledge the potential harm of treating based solely on urinalysis, and we emphasize that diagnostic decisions should always be made in conjunction with a full clinical assessment and history taking. Based on our findings and the existing literature, we believe there is a valid rationale for performing urinalysis in this specific group—but this should not automatically lead to antibiotic treatment. Rather, it should contribute to the overall diagnostic reasoning, helping to make a UTI more or less likely. We must also remain willing to await culture results and refrain from treatment when an alternative explanation for the clinical presentation is plausible.*

*We elaborated further on this subject tin the discussion, see page 18-29, lines 329-342. ‘Urinalysis, performed more frequently in the intervention group, detected true infections in about 20% of cases. In nearly 10%, antibiotics were prescribed for what appeared to be asymptomatic bacteriuria. While clinical judgment likely informed these decisions, we did not monitor whether other signs of infection were present. Diagnosing a urinary tract infection (UTI) in older adults is complex, particularly in patients with NSC, as presentations are often atypical, such as confusion, falls, or reduced appetite, rather than classic urinary symptoms (24). Genitourinary infections are among the most frequent diagnoses in this group, which may justify performing urinalysis as part of the diagnostic work-up (3, 25). However, given the high prevalence of asymptomatic bacteriuria and the risk of false positives, urinalysis should always be interpreted in the broader clinical context (26). While our findings support routine urinalysis in this group, treatment decisions should not rely solely on these results. Instead, urinalysis should be one component of the overall diagnostic reasoning, helping to assess the likelihood of a UTI. It is also important to wait for culture results and consider alternative explanations for symptoms to avoid unnecessary antibiotic use.’*

Comment 3.4: You mention that calcium and TSH should be excluded due to low yield of abnormal results with clinical significance, however there is a similar low yield for ALAT (3%). Can you explain why you have not suggested this be excluded? In the UK LFTs are not routinely measured in urgent care unless clinically felt necessary (and GGT only rarely according to clinical need) so this data is very helpful in this setting and could help reduce healthcare costs.

*Reply 3.4: Thank you for this valuable comment. You are correct in noting that ALAT showed a similarly low yield of abnormal results (3%) as calcium and TSH. Based on this, we indeed recommend excluding ALAT from the standardized diagnostic panel for patients presenting with NSC. However, interpretation is complicated by the fact that gamma-glutamyl transferase (GGT) was elevated in 9% of cases. Since isolated GGT elevations often require contextual interpretation alongside transaminase levels, we propose using GGT as a screening tool, with re-evaluation of ALAT only when GGT is abnormal. This stepwise approach aims to balance diagnostic value with efficiency and aligns well with efforts to reduce unnecessary testing and healthcare costs.. See page 18, lines 313-324. ‘ALAT was abnormal in only 3.0% of cases in our study, consistent with findings from a U.S. study demonstrating a similarly low diagnostic yield of routinely ordered liver tests (23). ALAT is frequently included as part of automated biochemical panels, yet our results suggest its clinical utility in patients presenting with nonspecific complaints (NSC) is limited. Based on the low proportion of abnormal results, it may be reasonable to consider excluding ALAT from standardized diagnostic protocols for this population. However, interpretation is complicated by the fact that gamma-glutamyl transferase (GGT) was elevated in 9.0% of cases in our cohort. As isolated GGT elevations often require contextual interpretation alongside transaminase levels, we recommend using GGT as a screening parameter, with ALAT re-evaluated only when GGT is abnormal. In light of its limited added value, we suggest removing ALAT from the standardized panel at this stage. This approach warrants further evaluation and may be adjusted as broader evidence emerges.*

*Based on these findings, we recommend adhering to our prespecified laboratory work-up for NSC patients, while excluding standardized measurements of ALAT, calcium, and thyroid screening—unless medical history, physical examination, or other laboratory values indicate their relevance.’*

Comment 3.5: You mention that ECGs only rarely showed clinically significant abnormalities and should be used selectively, however in the data 16% of patients had a clinically significant abnormality, which I would consider frequent, rather than rare. I would suggest your data actually suggests ECG is very useful in this population - picking up abnormalities ion 1 in 6 patients, and should be routine in such cases.

*Reply 3.5: We appreciate this comment and agree that a 16% rate of clinically significant abnormalities on ECG is not insignificant. Our original phrasing may have understated the relevance of this finding. While our intention was to suggest a more targeted rather than blanket approach—given the broad use of ECGs in patients with minimal cardiovascular suspicion—we acknowledge that identifying a potentially important abnormality in 1 out of 6 patients supports the utility of routine ECG use in older adults with nonspecific complaints.*

*In response, we have revised the relevant section of the manuscript to more accurately reflect the diagnostic value of ECGs in this population, and to suggest their routine use may indeed be justified, especially given the non-invasive nature, low cost, and potential to detect clinically actionable findings. See page 20, lines 370-378. ‘Our findings showed that 16% of NSC-patients showed significant abnormalities on ECG. This indicates that the diagnostic yield of ECG in this population is relatively high, with the test revealing a potentially important abnormality in approximately 1 out of every 6 patients. Given the often vague and non-specific nature of NSC presentations and the frequent unreliability of history taking in this population, the ECG appears to be a valuable diagnostic tool that can uncover underlying cardiac conditions that might otherwise remain undetected. These results support the routine use of ECG in the initial assessment of patients with NSC, as it may contribute to earlier recognition of serious pathologies and guide further diagnostic or therapeutic steps.’*

Comment 3.6: The paper is otherwise well written and covers a very relevant and important area of healthcare. I suggest the above modifications to the conclusions/discussion to allow a more considered and clinically relevant interpretation of your results. Many thanks.

*Reply 3.6: Thank you for your positive feedback and for highlighting the relevance of our work. We appreciate your suggestions and have revised the Discussion and Conclusions sections to provide a more considered and clinically relevant interpretation of our findings. We believe these changes strengthen the overall contribution of the paper.*
