# Supplementary material for: Evaluation of diagnostic measurements in patients with non-specific complaints: A secondary analysis after implementation of a care-pathway in the emergency department
Source: PLoS One. 2025 Sep 9;20(9):e0331060. doi: 10.1371/journal.pone.0331060 (PMC12419613; doi:10.1371/journal.pone.0331060)
Supplement: Attachment — Submitted filename: Response_to_reviewers_auresp_2.docx [file pone.0331060.s004.docx]

Dear Dr. Bayer,

Thank you for your time and effort in reviewing our manuscript. We have carefully addressed the inconsistency highlighted by the reviewer and revised the sentence accordingly. Please see page 18, lines 332–334:
“Urinalysis, performed more frequently in the intervention group, detected bacteriuria in about 20% of cases. In nearly 10%, antibiotics were prescribed for what appeared to be asymptomatic bacteriuria.”

We believe that the updated manuscript now meets the standards for publication in PLOS One.

On behalf of all authors,
Marleen van der Velde

**Reviewer #2: Many thanks for your updates and revised recommendations, I feel this is a good summary of your outcomes and that the revised document draws sensible conclusions. I would like however if the phrase "true infections" (line 341) could be altered with regards to the positive urine cultures - the 19.9% of cultures with a pathogen identifed represent bacturia only and can only be classed as true infections if they are correlated with symptoms (as described in the revised text). Could this phrase please be amended to reflect this)**

*Reply: Thank you for your time and effort in reviewing our manuscript. We have carefully addressed the inconsistency highlighted by you and revised the sentence accordingly. Please see page 18, lines 332–334: “Urinalysis, performed more frequently in the intervention group, detected bacteriuria in about 20% of cases. In nearly 10%, antibiotics were prescribed for what appeared to be asymptomatic bacteriuria.”*

*We believe that the updated manuscript now meets the standards for publication in* PLOS One.

*On behalf of all authors,
Marleen van der Velde*
